# Factors influencing trainers’ feedback-giving behavior: a cross-sectional survey

**DOI:** 10.1186/1472-6920-14-65

**Published:** 2014-04-01

**Authors:** Elisabeth AM Pelgrim, Anneke WM Kramer, Henk GA Mokkink, Cees PM van der Vleuten

**Affiliations:** 1 Department of Primary Care and Community Care, Radboud University Nijmegen Medical Centre, Postbus 9101, Huispostnummer 117, 6500 HB Nijmegen, the Netherlands; 2 Department of Educational Development and Research, Faculty of Health, Medicine and Life Sciences, Maastricht University, P.O. Box 616, 6200 MD Maastricht, the Netherlands

**Keywords:** Feedback-giving, Neuroticism, Task perception, Big five

## Abstract

**Background:**

The literature provides some insight into the role of feedback givers, but little information about within-trainer factors influencing ‘feedback-giving behaviours’. We looked for relationships between characteristics of feedback givers (self-efficacy, task perception, neuroticism, extraversion, agreeableness and conscientiousness) and elements of observation and feedback (frequency, quality of content and consequential impact).

**Methods:**

We developed and tested several hypotheses regarding the characteristics and elements in a cross-sectional digital survey among GP trainers and their trainees in 2011 and 2012. We conducted bivariate analysis using Pearson correlations and performed multiple regression analysis.

**Results:**

Sixty-two trainer-trainee couples from three Dutch institutions for postgraduate GP training participated in the study. Trainer scores on ‘task perception’ and on a scale of the trait ‘neuroticism’ correlated positively with frequency of feedback and quality of feedback content. Multiple regression analysis supported positive correlations between task perception and frequency of feedback and between neuroticism and quality of feedback content. No other correlations were found.

**Conclusion:**

This study contributes to the literature on feedback giving by revealing factors that influence feedback-giving behaviour, namely neuroticism and task perception. Trainers whose task perception included facilitation of observation and feedback (task perception) and trainers who were concerned about the safety of their patients during consultations with trainees (neuroticism) engaged more frequently in observation and feedback and gave feedback of higher quality.

## Background

Research has shown that learning from feedback is a complex process influenced by individual and cultural factors [1]. Within the process of giving and receiving feedback the literature provides some insight in three key elements: the feedback giver, the feedback recipient and the feedback content. Two reviews on the *feedback content*[2,3], each covering over one hundred articles and book chapters, showed that feedback is best aimed at a problem or task, at the process or at self-regulation but never at a personal trait of the recipient. Feedback should also enable comparison with an established standard. Psychological studies and recent medical education research [4-12] have shown that the role of the *feedback recipient* is an active one with recipients seeking information explicitly, for example by asking a trainer: ‘how am I doing’, or implicitly by making use of feedback intended for others [6,8]. Feedback-seeking behaviour is affected by personal and contextual factors [5-7] and feedback recipients decide whether to accept and use feedback [10], based on factors like self-reflection on performance [13], reflection on feedback [14] and the perceived credibility of the feedback source [10]. A literature search on the feedback context, the *feedback giver* in particular, however, yielded far fewer results than searches on the *feedback content* and the *recipient.*

In the present study we focus on the feedback giver. In the only study we found on feedback-giving behaviour, Adams reported that feedback-giving behaviour in the US Army helicopter training school was influenced by a positive affect of trainers, with trainees who were well liked by trainers receiving less positive and less specific feedback. The author concluded therefore that popular trainees should be especially vigilant in pursuing feedback necessary for their personal development [15]. Evidence from studies on feedback seeking showed that benefits of feedback as perceived by trainees depended on the trainer [9,11,12]. Trainers who combined a supportive (involved and accessible) and instrumental (focusing on rules and responsibilities) supervisory style were more successful in convincing residents of the value of directly asking for feedback [9,11,12]. Additional evidence from an earlier study [16] showed that trainers who were active feedback givers were able to overcome trainees’ feedback averse behaviours. Despite quite a few studies on the role of the feedback giver, not much is known about factors influencing ‘feedback-giving behaviour’, although an interview study by Kogan et al. [17] showed greater perceived ease of giving feedback among trainers who were more self-confident about feedback giving.

To contribute to the literature on feedback giving, we investigated the impact of personal characteristics of feedback givers on feedback-giving behaviour, specifically on feedback after observation of single patient encounters in postgraduate GP training. This feedback process encompasses three elements: *organization* (frequency), the *quality of feedback content* (does the feedback adhere to directives about the ‘feedback content’) and *consequential impact* (does the trainee use the feedback to determine and pursue learning goals and link present and previous feedback) [16]. Based on the literature on trainer effects on these three elements [13,16,18] and on the researchers’ experiences, we hypothesized six correlations between trainer characteristics and elements of the feedback process.

### Hypothesis 1

Based on a study by Kogan et al. into effects of trainers’ self-confidence [17], we formulated a hypothesis on self-efficacy, i.e. the belief in one’s ability to succeed in a specific situation [19], in this case the ‘preconditions’ of the feedback process, i.e. arrangements to facilitate observation and feedback and support trainees in using feedback. We hypothesized that trainers with strong self-efficacy are more inclined to give feedback *(frequency),* give better feedback *(quality of content)* and are better able to convince trainees to use feedback for improvement *(consequential impact)*.

Hypothesis 1: A trainer’s high sense of self-efficacy (concerning preconditions for the feedback process) is positively correlated with the three elements of the feedback process, frequency, quality of content and consequential impact.

### Hypothesis 2

Previous research showed that giving feedback is a core characteristic of competent trainers [20,21]. It seems plausible that trainers who do not consider it to be their task to create favourable preconditions for feedback should have a negative effect on the three elements of the feedback process. In line with this reasoning we proposed the following hypothesis:

Hypothesis 2: A trainer who sees it as his or her professional task to create positive preconditions for the feedback process (positive task perception) shows better feedback-giving behaviour in terms of frequency, quality of content and consequential impact.

### Hypotheses 3, 4, 5 and 6

The Big Five refers to a taxonomy of personality traits comprising five domains: neuroticism, extraversion, conscientiousness, agreeableness and openness to experience [22,23]. Based on a study by Krasman of the impact of these traits on *feedback-seeking behaviour*[7]*,* we formulated four hypothetical relationships between the Big Five and elements of *feedback-giving behaviour*.

*Neuroticism* refers to a person’s emotional stability. Krasman demonstrated that people with a more neurotic personality tend to seek more feedback, probably to alleviate their strong sense of insecurity [7]. A similar effect might be seen in trainers who give more feedback to compensate for their feelings of insecurity about for example entrusting the care of their patients to a trainee. Using a similar line of reasoning, we hypothesized that more neurotic trainers should be more inclined to make sure their trainees do use feedback (consequential impact). Neuroticism could have a negative effect on the content of feedback because people with a more neurotic personality are more easily frustrated, irritable and prone to react violently [23]. These considerations resulted in the following hypothesis:

Hypothesis 3: Compared to trainers with a fairly stable emotional make-up, trainers with a neurotic personality score higher on frequency and consequential impact of feedback and lower on the quality of the content of the feedback process.

*Extraverted* people are very sociable, enthusiastic and action-oriented; they like to talk and be the centre of attention in groups. Although introverted people too may be very active and energetic, they are less sociable. Krasman demonstrated that extraverted people are inclined to seek more feedback [7]. Because feedback giving is an interpersonal activity, extraverted trainers may engage more intensely in feedback giving with a positive effect on frequency and consequential impact. We saw no reason to expect extraverted trainers to give better feedback (quality).

Hypothesis 4: The personal characteristic extraversion correlates positively with the frequency and consequential impact of the feedback process.

Although people who rank high on *agreeableness* are interested in other people’s concerns, Krasman found no correlation between agreeableness and feedback-seeking behaviour. Since feedback-giving behaviour is related to an interest in other people’s concerns, we considered it nevertheless plausible that agreeableness should correlate positively with frequency, consequential impact and quality of feedback.

Hypothesis 5: The personal characteristic agreeableness is positively correlated with the elements frequency, quality and consequential impact of the feedback process.

People with a *conscientious* personality tend to prefer planned to spontaneous behaviour, have strong self-discipline and a strong sense of duty and aim for achievement against certain standards or outside expectations. Conscientious people are intent on performing their tasks properly and Krasman found that they sought more feedback [7]. It therefore seemed plausible that conscientious trainers should score high on the three elements of feedback-giving behaviour.

Hypothesis 6: The personal characteristic conscientiousness is positively correlated with the frequency, quality of content and the consequential impact of the feedback process.

*Openness to experience* reflects the degree to which people enjoy rich, varied and novel experiences. Krasman was unable to establish a correlation between openness and active feedback-seeking behaviour but did find a correlation with passive feedback seeking. However, considering that feedback giving is an active behaviour and not directly linked to rich, varied and novel experiences, we did not hypothesize a relationship between openness to experience and elements of feedback giving behaviour. Table 1 presents an overview of the hypotheses we tested.

**Table 1:** Hypothesized relationships of personality traits and elements of feedback-giving behavior

	**Feedback-giving behavior**		
	**Frequency**	**Quality of content**	**Consequential impact**
Positive self-efficacy	+	+	+
Positive task perception	+	+	+
High on neuroticism	+	-	+
High on extraversion	+		+
High on agreeableness	+	+	+
High on conscientiousness	+	+	+

## Methods

To test the hypotheses we conducted a cross-sectional survey by administering digital questionnaires to trainer-trainee couples. Trainers and trainees answered different questionnaires. The trainer questionnaire contained questions about six independent variables: self-efficacy, task perception, neuroticism, extraversion, agreeableness and conscientiousness, and the trainee questionnaire contained questions about the three dependent variables: frequency, quality of content and consequential impact of the feedback process.

### Study context

GP trainees in the Netherlands spend much of their postgraduate training working in a general practice where they are supervised by the same GP trainer for a prolonged period of time (around one year). This is different compared to the hospital context where a trainee may have several supervisors. The trainer can observe the trainee during patient consultations and give feedback on performance. They work in the practice four days a week, and one day per week attend a day release program at the university. Although they work independently most of the time, trainees can ask their trainer for help and advice and arrangements can be made for direct or video observation of a patient consultation followed by feedback.

### Participants

Each of the eight University Medical Centres in the Netherlands offers a postgraduate training program for general practice delivered by the local GP training institution. Within the framework of a faculty development program, trainers and trainees from the institutions at Groningen, Utrecht and Rotterdam participated in our study. Before the start of the faculty development program we gathered information about giving feedback among the participants. These three institutions were selected for the faculty development program partly because of their geographical location in the north, middle and west of The Netherlands including both urban and rural settings and partly based on practical reasons (fitness of the development program in their local program). We asked first-year trainees and their trainers to fill in a digital questionnaire in the trainees’ second or third month of training. During the first months of training trainees are usually observed fairly frequently, because trainers are eager to gauge their competence. We sent an invitation to participate in the study to all 248 trainees who had started training in September 2011 or March 2012 and their trainers (Groningen 64, Utrecht 98 and Rotterdam 86). 183 trainer-trainee couples signed for informed consent (Groningen 37, Utrecht 73 and Rotterdam 73). Two groups of trainer-trainee couples, one starting training in September 2011 and the other in March 2012, were asked to participate in the survey at the end of October or in November 2011 and at the end of April or in May 2012, respectively. Halfway these periods non-responders received a digital reminder. The ethics review board of the Dutch Association of Medical Education approved the study. All participants signed for informed consent.

### Questionnaire

All four researchers (EP, AK, HM and CvdV) contributed to the development of the questionnaires. In July 2011 the questions were scrutinized by a group of experts/researchers consisting of two educational scientists, two GPs and an obs/gyn specialist. The feedback from the experts was used to adjust the questionnaires. The questionnaire was never used before but based on the literature [16,17,22,24] and on expert knowledge which provides some face validity. The questionnaire is attached in Additional file 1: Tables S1 to S3.

#### Independent variables

The trainer questionnaire contained questions about self-efficacy and task perception and questions from parts of a big-five questionnaire [22]. Task perception and self-efficacy were measured by three questions relating to preconditions for the feedback process (Additional file 1: Table S1). The questions about the personality traits were based on the Dutch version of the revised NEO Personality Inventory (NEO PI-R) and NEO Five-Factor Inventory (NEO-FFI). There were seven questions for each personality trait (Additional file 1: Table S2), which were mixed and anchored to either of the two extremes of a trait.

#### Dependent variables

Questions about trainers’ feedback-giving behaviour in relation to the three dependent variables (frequency, quality of content and consequential impact) were put to trainees because we were more interested in trainees’ perceptions of the feedback they received than in trainers’ perceptions of the feedback they provided. Six questions about ‘frequency’ (Additional file 1: Table S3) asked about the number of observations and the time spent on observations and feedback discussions. By multiplying the number of observations by the mean number of minutes per observation we obtained the total time (in minutes) spent on observation and feedback during the first two months of training. We adapted the measure of ‘*quality of content’* from the study by Adams [15] to the setting of our study and translated the questions into Dutch. This resulted in twelve questions with five-point Likert scales. Based on recommendations from a review by Archer [24], we measured ‘*consequential impact’* using three questions about the linkage between feedback and trainees’ learning goals, the possibility for trainees to reflect and linkage of new with earlier feedback, all with a five-point Likert scale (Additional file 1: Table S3).

### Analysis

For the continuous variables we calculated means and SDs. The scores of negatively formulated items were recoded. For self-efficacy, task perception, quality of content and consequential impact, the Likert type scores are presented as percentages after dichotomization (1–3 (fully) disagree or partly disagree versus 4 and 5 (fully) agree). We used the dichotomization only to show the distribution of data. Scores were aggregated per item by calculating the mean sum score for the original data, i.e. without dichotomization. To test for internal consistency we calculated Cronbach’s alpha and conducted factor analysis for the Likert scores. The six questions about frequency of feedback were also put to the trainers, and trainers’ and trainees’ answers were analyzed using Pearson correlations. The trainers’ answers were only used to check the quality of the data. Moreover, we used the data of trainees whose trainer did not respond to check for differences in (trainer) responders and (trainer) non-responders.

After bivariate analysis to test the hypotheses using Pearson correlation, we performed multiple regression analysis. The variables neuroticism and task perception were included. An alpha level of .05 was considered to be significant.

## Results

### Descriptive statistics

**Figure 1:**
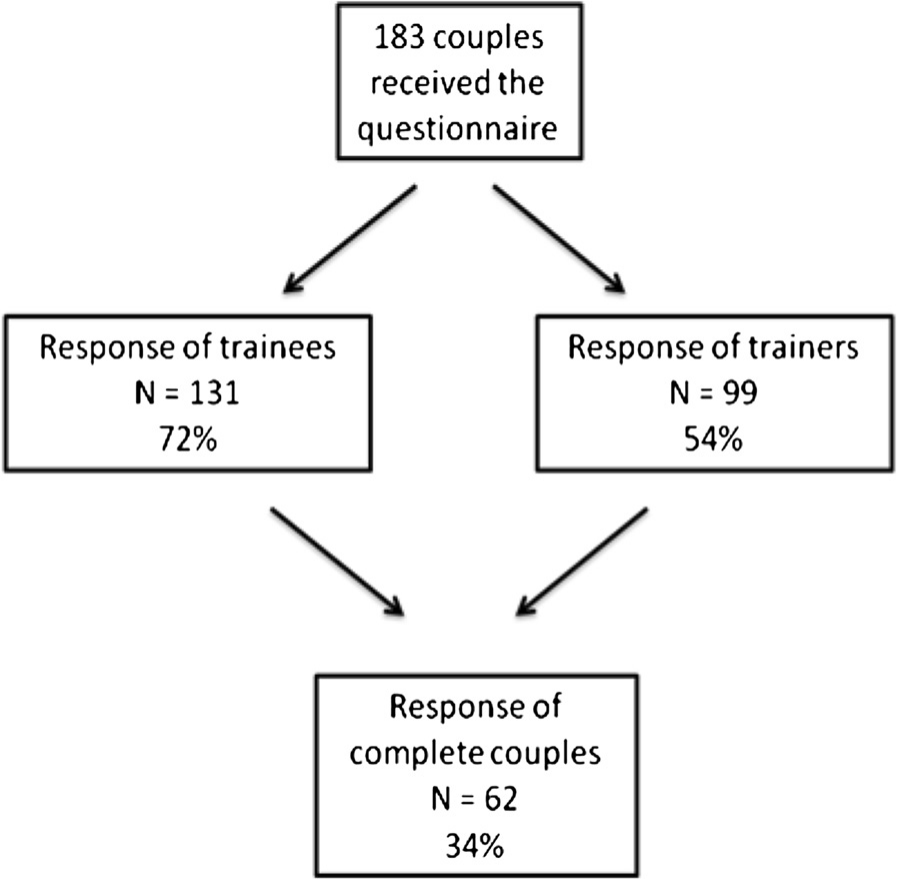
Flowchart of response rates.

Of the total of 248 trainer-trainee couples that were eligible for inclusion in the study, 183 gave informed consent and received questionnaires. Sixty-two couples (34%) completed the questionnaires (Groningen 43%, Utrecht 37%, Rotterdam 26%). The rather low response rate is attributable to the use of trainer-trainee couples as the unit of analysis which meant exclusion of a couple if data for one member was missing (Figure 1).

To check for differences in (trainer) responders and (trainer) non-responders we used the data of the trainees’ questionnaire and compared the results of trainees whose trainer did not respond with the answers of trainees whose trainers did respond. We used the ANOVA to analyse whether differences between these two groups were statistical significant. Table 2 shows that there were no significant differences in the data that we were able to analyse (frequency, quality of content and consequential impact).

**Table 2:** Average values of trainer responders and non-responders and level of significance between them (ANOVA)

	**Trainer responders (N = 62)**	**Trainer non-responders (N = 69)**	**Sign. (ANOVA)**
Frequency	678*	611*	.55
Quality of content	3.57	3.63	.48
Consequential impact	3.94	3.85	.24

*minutes over two months.

### Independent variables

Table 3 shows the descriptive statistics for the independent variables. Because of the low alpha for task perception we performed factor analysis, which showed that the questions represented one component. So, despite relatively low internal consistency, the items appeared to be related to one construct. The last column shows that at least three-quarters of the respondents did not agree that it was their task (task perception) or felt confident to ensure preconditions for feedback (self-efficacy), such as arranging for observation and feedback or supporting trainees in translating feedback into learning goals.

**Table 3:** Descriptive statistics for the independent variables

	**Mean**	**Minimum**	**Maximum**	**SD**	**α**	**Factor**	**4 or 5 on Likert scale (%)**
Self-efficacy	3.43	2.33	5.00	.53	.71		19.4
Task perception	3.50	2.33	4.67	.49	.30	1	24.2
Neuroticism	2.22	1.00	3.29	.44	.78		
Extraversion	3.60	2.57	4.71	.53	.78		
Agreeableness	3.89	2.71	4.71	.35	.63		
Conscientiousness	3.52	2.57	4.57	.52	.81		

### Dependent variables

Two researchers (EP and HM) examined extreme scores on the variable frequency, because due to misinterpretation of questions 2, 3, 5 and 6 (Additional file 1: Table S3) some participants had multiplied the answers to questions 1 or 4 by the average number of minutes. When both researchers were certain this had happened, the mistake was corrected. The results show huge variation in the number of minutes of observation and feedback over two months. The maximum of 3090 minutes means in practice 1.6 hours of observation and feedback per day (based on 4 days a week for 8 weeks). There were significant correlations between the answers of trainees and their trainers.

The alpha for quality of content was high. Factor analysis performed because of the low alpha for consequential impact showed that the items appeared to represent one construct. The last column in Table 4 shows that only a minority of trainees gave high scores on quality of the content of feedback, whereas almost 70% indicated that their trainers took steps to ensure the consequential impact of feedback.

**Table 4:** Descriptive statistics for the dependent variables

	**Mean**	**Minimum**	**Maximum**	**SD**	**Correlation with answers given by trainers**	**α**	**Factor**	**4 or 5 on Likert scale (%)**
Frequency	678*	70*	3090*	573*	.36†			
Quality of content	3.57	2.25	4.85	.44		.86		17.7
Consequential impact	3.94	2.67	4.67	.42		.47	1	69.4

*minutes over two months.

†P < .01.

### Correlation of dependent and independent variables

We found four significant correlations between dependent and independent variables (Table 5). Task perception and neuroticism showed positive correlations with frequency and quality of feedback.

**Table 5:** Significant correlations between independent and dependent variables

	**Feedback-giving behavior**		
	**Frequency**	**Quality of content**	**Consequential impact**
Positive self-efficacy			
Positive task perception	.30*	.34†	
High on neuroticism	.33†	.31*	
High on extraversion			
High on agreeableness			
High on conscientiousness			

*P < .05.

†P < .01.

To examine the potential interdependence of these correlations we performed multiple regression analysis. This showed that for frequency of feedback the independent variables task perception and neuroticism were correlated. As only the influence of task perception remained significant, the correlation between frequency and neuroticism depended on task perception. For quality of content things were the other way round with task perception depending on neuroticism.

## Discussion

The purpose of this study was to contribute to the literature on feedback-giving behaviour by adding insights into factors within the person of the feedback giver. Task perception and the personality trait neuroticism were found to influence two elements of the feedback process: the frequency of feedback and the quality of feedback content. The results appear to support the conclusion that trainers who consider it to be their task to create favourable conditions for observation and feedback, are likely to show a higher frequency of feedback as well as better quality of feedback content. This is in line with hypothesis 2, but the results provide no evidence for the postulated effect on consequential impact. The finding that task perception influences frequency and quality of feedback, is interesting in light of the finding that over 75% of the participating trainers disagreed with the statement that creating preconditions for observation and feedback was part of their task. This may offer a key to improving observation and feedback in general practice training. Earlier research showed that trainers who take an active attitude towards feedback giving are able to activate inactive trainees [16]. The present results, however, indicate that a positive task perception is prerequisite for trainers to observe and provide feedback more frequently.

Our results indicate a relationship between a neurotic personality of the trainer and frequency of observation, implying that emotionally stable trainers observe less frequently. This is in line with our hypothesis and with the literature on feedback seeking. Krasman demonstrated that neurotic persons seek more feedback, probably to alleviate a sense of insecurity [7]. Our results add to this that trainers with a more neurotic personality tend to observe more frequently. A possible explanation could be that they may feel insecure leaving their patients in the care of trainees and are eager to ensure that their patients are safe. As a consequence the relative frequency of observation and feedback by emotionally stable trainers is lower. It should be noted that the correlation between neuroticism and the quality of the content of feedback turned out to run in opposite to the direction we had hypothesized. Our hypothesis stated that neurotic people are easily frustrated, irritable and prone to react violently [23] and that this detracts from the quality of feedback, whereas emotionally stable trainers provide better quality feedback. The findings, however, turned out to be the other way round. Apparently, feelings of insecurity had a positive effect on the quality of feedback while more stable personalities seemed more likely to leave matters to others. We found no evidence for an impact of neuroticism on consequential impact. The results support the conclusion that trainers with a more neurotic personality tend to give more feedback and that this feedback is of better quality.

Task perception and neuroticism were found to be interdependent. In other words, of the trainers with a high task perception a large group had a more neurotic personality or alternatively of the trainers with a more neurotic personality a large group had a high task perception. Feelings of insecurity might be involved too. Whereby, trainers with a more neurotic personality may be more inclined to comply with recommendations regarding observation from the training institution.

The results do not support effects of self-efficacy, extraversion, agreeableness or conscientiousness of trainers on elements of the observation and feedback process. This means that the results support neither our hypotheses nor results from the (feedback-*seeking)* literature [7,17]. This may be explained by the fact that feedback seeking and feedback giving are two different concepts relating to activities that are driven by different purposes. In feedback seeking the focus is on the person seeking feedback, who is intent on developing or demonstrating their own performance [9]. In feedback giving on the other hand the focus is on the recipient of the feedback, while the feedback is usually provided by someone in a professional capacity. The differences between our results and those of Kogan et al. [17] in relation to self-efficacy may be due to differences in operationalization. These differences warrant further research to clarify the potential effects of self-efficacy.

Recent research in the field of organizational psychology concluded that the feedback orientation of employees accounts for a substantial portion of the variance in the quality of coaching relationships between employees and their supervisors. In addition, empirical evidence supported a link between the coaching relationship and actual coaching behaviours, with perceptions of the coaching relationship accounting for significant variance in reports of actual coaching behaviour [25]. This shows that feedback recipients play a prominent role in the way feedback is given. Based on our findings and the literature on feedback seeking [9,11,12,26] we can conclude that feedback-seeking and feedback-giving behaviours constitute a highly complex phenomenon in which several actors and factors are interacting in complex and intricate patterns. Moreover, the process appears to be shaped by the hierarchical nature of the relationship between feedback seeker and feedback giver, whether this is a trainee and a trainer, an employee and a supervisor or otherwise.

The findings from this study are affected by limitations of the research method. Our failure to find any correlation between consequential impact and independent variables may be explained by the low alpha of consequential impact and the high scores on this variable from almost 70% of the participants, suggesting that a large majority of the trainees were convinced that their trainers took steps to further the consequential impact of feedback. These high scores, however, make it difficult to measure correlations with independent variables. Additionally, the high scores on consequential impact run counter to previous research showing that only 12% of trainee-trainer couples formulated an action plan based on feedback on observed performance [18]. A possible explanation may be that although no action plan was formally recorded, trainees nevertheless perceived that their trainers expected them to act upon feedback they had received. It is also important to notice the low alpha for the explanatory variable task perception and the wide range of the scores on this variable while factor analysis showed that it was one component. A final limitation is the rather low response rate due to the requirement that answers had to be obtained from both members of trainer-trainee couples. Unfortunately, despite satisfactory response rates for trainees and trainers separately, many responses could not be analyzed because data for one member of a trainer-trainee couple was missing. Based on the data we had from trainees with a non-responding trainer we could conclude that there was no significant difference between trainers responding and non-responding with regard to the dependent variables. We have no information about possible differences between trainers responding and non-responding with regard to the independent variables (personality of the trainer).

One of the strengths of this study is that participants were recruited from three different institutions for general practice, which strengthens the generalizability of the results to all programs for postgraduate training in general practice in the Netherlands. Further research should focus also on the feedback process towards the end of the training period of a trainer-trainee couple. Our study focused on the beginning of the period. Possibly the way of giving and receiving feedback changes when the relationship evolves. In addition to the relationship is also the professional culture of influence on feedback [1]. Further research could also focus on differences between the GP-setting and the hospital setting, or even with settings outside medical education.

Our findings have practical implications as well. Because of its influence on feedback-giving behaviour, task perception should be a focus of training for trainers. Faculty development activities in postgraduate medical education often focus on (didactic) skills. The results suggest, however, that it may not be so much deficiencies in didactic skills but rather shortcomings in task perception that prevent trainers from engaging in observation and feedback. Our finding about the influence of the personality trait ‘neuroticism’ is more difficult to implement in practice. Perhaps it can be used as a variable in selection procedures or as a reflection variable in faculty development programs.

This study expands on the literature on feedback giving by adding two factors that impact on feedback-giving behaviour: trainers’ neuroticism and task perception.

## Conclusions

This study contributes to the literature on feedback giving by revealing factors that influence feedback-giving behaviour, namely neuroticism and task perception. Trainers whose task perception included facilitation of observation and feedback (task perception) and trainers who were concerned about the safety of their patients during consultations with trainees (neuroticism) engaged more frequently in observation and feedback and gave feedback of higher quality.

### Ethical approval

The study was approved by the ethics review board of the Dutch Association for Medical Education.

## Competing interests

The authors declare that they have no competing interests.

## Authors’ contributions

EAMP performed this research at the Department of Primary Care and Community Care, Radboud University Nijmegen Medical Centre, The Netherlands. She has a PhD in medical education. Her PhD thesis concerns feedback based on direct observation of clinical encounters in workplace-based assessment in the GP-training setting. AWMK, PhD, is a senior researcher and leader of the research programme ‘profesional development in general practice’, at the Department of Primary Care and Community Care, Radboud University Nijmegen Medical Centre, The Netherlands. She also works as a general practitioner in Utrecht. HGAM, PhD, is methodologist at the Department of Primary Care and Community Care, Radboud University Nijmegen Medical Centre, The Netherlands. CPMVV, PhD, is a Professor of Education, Chair of the Department of Educational Development and Research, Scientific Director of the School of Health Professions Education (SHE) at Maastricht University, The Netherlands. He holds honorary appointments in the University of Copenhagen (Denmark), King Saud University (Riyadh) and Radboud University (Nijmegen). All authors read and approved the final manuscript.

## Pre-publication history

The pre-publication history for this paper can be accessed here:

http://www.biomedcentral.com/1472-6920/14/65/prepub

## Supplementary Material

Additional file 1: Table S1 Items for the independent variables self-efficacy and task perception. **Table S2.** Items for the independent variables neuroticism, extraversion, agreeableness and conscientiousness. **Table S3.** Items relating to the dependent variables. Click here for file
