# Supplementary material for: Factors influencing trainers’ feedback-giving behavior: a cross-sectional survey
Source: BMC Med Educ. 2014 Apr 1;14:65. doi: 10.1186/1472-6920-14-65 (PMC4230419; doi:10.1186/1472-6920-14-65)
Supplement: Additional file 1: Table S1 — Items for the independent variables self-efficacy and task perception. Table S2. Items for the independent variables neuroticism, extraversion, agreeableness and conscientiousness. Table S3. Items relating to the dependent variables. [file 1472-6920-14-65-S1.doc]

**Table S1. Items for the independent variables self-efficacy and task perception**

| Measure | Sample item |
| --- | --- |
| Self-efficacy | Indicate to what extent you consider yourself able to:  1. Arrange for observation of a patient consultation performed by your trainee.  2. Schedule time for a feedback discussion after an observation.  3. Ensure that the trainee translates feedback into learning goals, in other words ensure that your trainee actually uses the feedback you give to enhance his/her learning.  (Answers on 5-point Likert-scale: 1= not at all; 5= totally) |
| Task perception | Indicate to what extent you consider the following activities to be a part of your tasks as a trainer:  1. Arrange for observation of a patient consultation performed by your trainee.  2. Schedule time for a feedback discussion after an observation.  3. Ensure that the trainee translates feedback into learning goals, in other words ensure that your trainee actually uses the feedback you give to enhance his/her learning.  (Answers on 5-point Likert-scale: 1= not at all; 5= totally) |

**Table S2**. Items for the independent variables neuroticism, extraversion, agreeableness and conscientiousness.

| Measure | Sample item |
| --- | --- |
| Neuroticism | I get upset easily  I tell myself that I am in trouble  I always see a ray of hope  I always fear for the worse  I quickly put aside setbacks  I can take a few blows  I quickly rack my brains over something |
| Extraversion | I make people smile  I keep in the background  I avoid company  I love big parties  I am afraid of new encounters  I start conversations  I prefer to be on my own |
| Agreeableness | I make efforts for other people  I take into account the interests of others  First of all, I think about myself  I take into account the feelings of others  I use others for my own goals  I impose my will on others  I respect other people’s opinion |
| Conscientiousness | I do things without planning  I do things at the last minute  I make my work on time  I work in a fixed pattern  I want everything exactly right  I leave my work unfinished  I am always well prepared |
|  | All answers on 5-point Likert-scale: 1 = this totally applicable to me;  5 = this is totally not applicable to me |

**Table S3. Items relating to the dependent variables**

| Measure | Sample item |
| --- | --- |
| Feedback-giving behaviour – frequency | 1. How many times were you observed (live or via video) during a consultation you performed in the last two months?  2. How long were you observed on these occasions? Give the average time in minutes, excluding the time for the feedback discussion.  3. Give the average time in minutes for the feedback discussion.  4. How many times were you observed during a home visit or night shift in the last two months?  5. How long were you observed on these occasions? Give the average time in minutes, excluding the time for the feedback discussion.  6. Give the average time in minutes for the feedback discussion.  (Answers in numbers [number of times or minutes]) |
| Feedback-giving behaviour – quality of content | 7. When I have performed poorly, my trainer provides detailed information about my performance.  8. When I have performed poorly, my trainer tells me specifically which aspects of my performance are in need of improvement.  9. When I have performed well, my trainer provides detailed information about my performance.  10. When I have performed well, my trainer tells me specifically which aspects of my performance were performed well.  11. I would like to receive more detailed feedback from my trainer.  12. After a discussion with my trainer I do not know exactly what I did well.  13. After a discussion with my trainer I do not know exactly what I did wrong.  14. My trainer tells me when I perform well.  15. My trainer gives me compliments.  16. My trainer tells me when I do not perform well.  17. My trainer expresses dissatisfaction when I do not perform well.  18. My trainer tells me when I have done something wrong.  (Answers on 5-point Likert-scale: 1 = never; 5 = always) |
| Feedback-giving behaviour – consequential impact | 19. The feedback I receive relates to my personal learning goals.  20. I get the opportunity to respond to feedback I receive.  21. The feedback I receive is linked to feedback I received on previous occasions.  (Answers on 5-point Likert-scale: 1 = never; 5 = always) |
